# General practitioners’ knowledge and practice in consultations with (potential) torture victims: a qualitative pilot study from Norway

**DOI:** 10.1080/02813432.2024.2404054

**Published:** 2024-09-13

**Authors:** Abinaya Anpalagan, Hanna Fesseha, Anette Bringedal Houge

**Affiliations:** a Faculty of Medicine, University of Oslo, Oslo, Norway; b Postdoctoral Researcher at Department of Interdisciplinary health research, Faculty of Medicine and Associate Professor at Department of Criminology and Sociology of Law, Faculty of Law, University of Oslo, Oslo, Norway

**Keywords:** Torture, primary healthcare, general practitioners, identification, refugee health, Norway

## Abstract

**Background:**

According to the UN Committee Against Torture, all state parties to the Torture Convention have a responsibility to meet the rehabilitation needs of torture victims who have sought asylum within their borders. General practitioners (GPs) can play a crucial role in identifying torture victims and securing rehabilitation when needed. There is a pressing knowledge gap on the knowledge and practices of GPs vis-à-vis potentially tortured patients, and an urgent need for research that investigates GPs’ practices of identification, referral, and rehabilitation – in Norway and beyond. This article presents an exploratory qualitative pilot study that investigates the experiences of GPs in Oslo vis-à-vis this patient group.

**Methods:**

Semi-structured interviews with five experienced GPs in the greater Oslo area. Interview data was analyzed through thematic analysis and discussed within a theoretical framework seeing GPs as street-level bureaucrats.

**Results:**

Issues that emerged in the pilot involve a consistent professional confidence and a particular concern for victims of sexual violence and sexualized torture. The pilot also found a troubling commonsensical reasoning about identification in the asylum process. The GPs asked for the re-establishment of specialist rehabilitation centers for refugees and torture victims to consult in case of need. Alas, the study also confirmed that GPs are a difficult profession to recruit for research.

**Conclusions:**

This study indicates that GPs are important actors in terms of identifying torture victims after resettlement, but that there are shortcomings in their training and knowledge, in the overall organization of the healthcare system, and in specialized healthcare, that limit prospects for rehabilitation.

## KEY POINTS

To what extent are torture victims met or identified by GPs in resettling contexts? This paper presents a pilot study designed to address this knowledge gap and prepare future studies. We found:

- GPs were confident in their abilities to identify and meet torture victims in a good way – and in the value that is patient-doctor continuity in general medicine.
- Troubling commonsensical reasoning about identification in the asylum process and in consultations.
- A lack of and need for qualified and available expertise to consult with and/or refer tortured patients to.

## Introduction

Torture is deliberately destructive [1]. Its health impacts are often severe and comprehensive [2]. Unidentified and untreated, torture injuries can limit victims’ prospects for integration and workforce participation, and the quality of life for both victims and their families [3]. Despite its absolute prohibition, torture prevails in an alarming number of states [4] and in every conflict generating refugees [5]. While there are no global estimates of the number of torture victims [6], we can safely state that many of the world’s 35 million refugees and asylum seekers [7] have, thus, survived torture [2]. The principle of non-refoulement obliges states to secure legal status and stay for migrants who are at risk of torture upon extradition [8]. Still, torture victims can hesitate to share their experiences in the asylum process for lack of trust in authorities, because of the suspicion they are met with, for fear of information transfer to the state responsible for their torture, and because it is a highly sensitive subject [9,10]. Case managers, on the other hand, might expect asylum seekers to share experiences with torture unsolicited as such experiences can strengthen their application [11]. Due to their gatekeeping function in the asylum system, case managers need to conduct credibility assessments, and may also meet applicants’ claims with skepticism and suspicion – a practice that is likely to restrain applicants’ perceived space for sharing sensitive experiences [12]. Yet, torture victims may be granted residence permit and legal stay for other reasons, e.g. humanitarian grounds, protection from other forms of persecution, or as refugees. It follows that many torture victims are resettled without having been identified as such by the state responsible for their healthcare [13].

**Table 1.** Characteristics of interviewees.

Interviewee #	Sex	Years as GP	List size
1	Male	20+	1400+
2	Male	20+	1100
3	Male	20+	1600+
4	Female	20+	1200
5	Female	20+	1000

The right to ‘as full rehabilitation as possible’ is part of the UN Convention against Torture [8]. According to the UN Committee Against Torture [14], state parties have a responsibility to meet the rehabilitation needs of torture victims who seek asylum within their borders. Rehabilitation, however, requires identification. While obliged to offer rehabilitation as per the Torture convention, several member states do not systematically inquire about torture experiences during asylum interviews, nor do they systematically transfer information to the municipal level in the resettlement process if torture victims are identified in asylum hearings or reception centers [11,15]. It follows that for resettled torture victims in these states, general practitioners (GPs) can play a crucial role in identifying victims and securing their right and access to rehabilitation when needed, as key providers of public healthcare and gatekeepers to specialized services [16]. In contrast to asylum hearings, general practitioners have the opportunity to develop trust over time, and a ‘close and continuous relationships with patients’, an feature of their job they tend to highlight as a primary motivation for their specialization [17]. As torture experiences are hard to share [18], this professional relationship can help victims open up. While substantial research has focused on issues to do with identification and medico-legal documentation in the asylum process [12,19], and on the quality of rehabilitation in specialized health services [20–23], there is a pressing knowledge gap pertaining to torture victims in primary healthcare services, and an urgent need for research that investigates GPs’ knowledge about torture and practices of identification, referral, and rehabilitation. To begin address this knowledge need, this article presents the findings of an Oslo-based pilot study designed to examine GPs’ knowledge and practices vis-à-vis potentially tortured patients.

In the following, we will first present the Norwegian context within which the study was conducted, and then introduce the theory of street-level bureaucracy (SLB) as the applied theoretical frame for the study. From there, the article continues in four main sections: First, we present our methodology, from recruitment and interviews, to presentation of interviewees and the analytic process. Because knowledge about GP practices is contingent on access to GPs in research, we also delve into the challenges we met in recruiting interviewees for this study. In the results section that follows, we present our findings in three thematic categories, based on the interviewees’ reflections on 1) Identification of torture victims, 2) The strengths of general practice, and 3) The organization of health care services. In the discussion, we address how the interviews revealed three corresponding concerns: 1) a problematic commonsensical reasoning, 2) an experience-based professional confidence under pressure, and 3) a downgrading of specialized health services for this patient group. In the article’s last section, we conclude with our reflection on the pilot’s limitations but, most importantly, implications, for future studies and practice.

### The Norwegian context

There are no permanent or systematic measures in place to identify torture injuries upon refugees’ arrival in Norway [11]. In fact, ‘the torture experience of asylum seekers is seldom addressed (…), hardly ever documented and rarely given particular consideration during the asylum assessment’ according to a recent study [24]. According to Weiss [24] this practice amounts to various forms of ‘ignorance that permeate the Norwegian immigration bureaucracy’ pertaining to torture victims in particular. With no systematic measures to identify torture victims upon refugees’ arrival, there are no comprehensive estimates of the number of refugees and migrants in Norway who have survived torture. 15 years ago, however, Fosse & Dersyd estimated that 31,000-35,500 refugees and migrants who had been granted asylum had been tortured before entry [25]. The refugee population has increased substantially since [26], particularly following the Arabic spring, warfare in Syria, and lastly, the Russian-Ukrainian war. It is safe to assume that the number of residents with torture experiences is markedly higher today and will continue to rise considering recent and ongoing warfare internationally. If applied as a conservative estimate of the present population, Fosse and Dersyd’s estimate suggests that each of Norway’*s* ∼5,000 GPs [27] would have an average of seven torture victims on their patient list.^1^ But how well prepared are Norwegian GPs to address the needs of this particular patient group?

The Norwegian Directorate of Health expect that all health personnel know symptoms of torture and how to follow up on identification, according to the international protocol for documenting torture injuries (the so-called Istanbul protocol). The national guidelines for *Health services for asylum seekers, refugees and family reunifiers* explicitly states that it is ‘crucial for the necessary health-related treatment and rehabilitation that the physical and psychological consequences of torture and other abuses that asylum seekers, refugees and family reunifiers have been exposed to are identified early’ [28]. In surveys, Norwegian GPs tend to emphasize continuity of care and the opportunity to develop trust and ‘close and continuous relationships with patients’ as primary motivations for their specialization [17]. On paper, thus, the GP scheme in Norway, according to which patients select their regular GP, offers a framework that facilitates good and lasting doctor-patient relationships, which can create a secure enough framework for patients to be able to reveal and talk about torture experiences.

However, and despite a gradual decrease in average patient list size, from approximately 1200 patients per list in 2005, to 1068 patients 15 years on [29], Norwegian GPs have an expansive workload with comprehensive and increasing administrative tasks, working 20 h above regular working hours [30]. International research points out how the increasing bureaucratization of health professionals’ workload – an international phenomenon – bolster medical professionals’ commitment to instrumental administrative values at the cost of core occupational ones [31]. Because of increasing workhours and a shift of workload from patient to administration in Norway, the GP scheme has come under severe pressure as GPs leave their specialization–amounting to what is commonly referred to as the GP-crisis [32].

Added to this, Norwegian general practice is based on a fixed price per consultation, on top of a basic allowance and reimbursement. It follows that ‘patient time’–key to trust and injury disclosure for torture victims [9]–is likely to be short in supply in a system that favors short consultations, straightforward health-challenges, and well-adjusted patients. Moreover, Norwegian GPs also report to know little about refugee health and refugee patients’ backgrounds [33,34].

In the case GPs identify torture injuries, there are no specialist rehabilitation centers they can consult with or refer patients to, in contrast to Norway’s Scandinavian counterparts^2^ where specialized, free of charge refugee and torture health and rehabilitation centers and clinics exist [15,35,36]. A possible referral must therefore be directed to ordinary specialist health services and patients must pay a set deductible for each consultation and treatment [10].

The Norwegian Health Care Services Act [37] emphasizes that health services are to provide universal, equal access and quality of services in primary care. Yet prior research on Norwegian GPs knowledge about and interest in refugee health [33,34] as well as the pressure at work present-day GPs report on, suggest that the overlap between ‘[health] *policy as written*’ and promised, and ‘[health] *policy as performed’* in everyday general practice is narrow for patients with torture injuries (paraphrasing Lipsky [38]).

### Theoretical frame

For theoretical framing, we have found street-level bureaucracy theory (SLB) to be particularly useful. SLB, a sociological theory coined by Michael Lipsky [39], pays attention to how so-called street level bureaucrats – frontline workers in public services – carry out and shape public policy in and through their practice [38,40]. Key to SLB-theory, is that it acknowledges how street-level bureaucrats operate between government and clients, and perpetually navigate between clients’ needs on the one hand, and available resources, regulations, and government policies on the other [41,42]. Moreover, the theory holds that most clients depend on the services of the street-level bureaucrats to get the help they need, and cannot afford or access private services elsewhere. Because the bureaucrats must process client caseloads en masse, they may develop a preference for easy clients for whom it is possible to perform an effective job. Such clients enables the bureaucrat to tick off clients from a seeming never-ending list of tasks (‘creaming off’ cases). Frontline workers may also become advocates for certain categories of clients that represent particular social vulnerabilities. By developing practice patterns that reduce the weight of individual decisions and modify the perception of clients into larger categories, street-level bureaucrats are pragmatic (or ‘street-smart’) [42]: they approach and adapt the reality of needs of clients according to their own work capacity, and the regulations and restraints of resources that they operate under. These coping strategies help them manage and overcome unmountable caseloads, but also renders them incapable of offering equal services to every client: they must – and do – prioritize. Additionally, the tasks they are set to solve for society are often governed by conflicting and ambiguous goals, rules and regulations [42]. The tensions involved and the structural limitations necessitate frontline bureaucrats’ exercise of professional discretion [43]. Discretionary practices and coping strategies may vary between offices because of institutional norms and in-house training and between individual front-line workers according to their workload, client base, and prioritization between different client categories. As a result, there can be a considerable *distance between law and policy as written, and law and policy as practiced*, as well as variation in-between front-line workers’ practices. SLB provides a useful vocabulary that can help address and understand GPs’ knowledge, discretional prioriti­zations, and potential coping strategies as front-line clinicians, navigating their patients’ (‘clients’ in SLB-vocabulary) needs in a context of limited resources (be they time, knowledge or other) [40]. In the presentation of results and discussion thereof, we will engage this SLB-terminology to address the practices of experienced GPs vis-à-vis potentially tortured patients.

## Methods

This study was developed as a pilot in preparation of a larger project on policies and realities for torture victims in primary health care in Norway, focusing on GPs knowledge about, practices pertaining to and perspectives on potentially tortured victims and their rights and needs. The recruitment of interviewees and data collection was undertaken by medical students Anpalagan and Fesseha at the University of Oslo. The data collection formed part of their medical thesis assignment and was carried out over three intervals throughout 2022-2023, each interval lasting four weeks. This article presents a thematic analysis of transcripts from interviews with five experienced GPs in Oslo who reflected on their own knowledge and practice vis-à-vis this patient population.

### Recruitment

Oslo has a population of 700 000,^3^ a quarter of which are immigrants [44], served by 566 GPs in 172 offices^4^ [44]. Considering the limited time available for data collection, and knowing that GPs are difficult to recruit for research purposes [45], we had an ambitious aim of recruiting 20 interviewees. As a pilot, we were also interested in testing recruitment effectiveness. We had a rigorous, flexible, and intensive recruitment plan to reach our target. With the ongoing war in Ukraine, an unprecedented refugee influx from the conflict, amidst reports of widespread war crimes, sexual violence, and torture, we considered our recruitment goal feasible. Unfortunately, the study confirmed that GPs are a particularly difficult profession to recruit, despite flexibility and perseverance.

The first step of recruitment was to reach out to the ten first GPs listed per Oslo district at the National online health services in Norway (see fn 3). GPs who were on leave, who substituted for another GP, or shared their list with a colleague were excluded along with those that had practiced less than 5 years. We first reached out to the offices of these 150 GPs per phone to inform about the study and the information letter we sent per email, and invite interviewees directly. Most of the health secretaries advised that we sent information per email due to a heavy workload and unavailability of the GP, some also refused participation directly on behalf of the physician, with reference to their workload. We never heard back from any of these 150 GPs. We therefore reached out per mail *and* phone to all 172 GP offices in Oslo. Three GPs agreed to participate. Next, we visited a number of offices, and left physical copies of the information letters with the health secretaries. We managed to speak directly with a few GPs, two of which agreed to take part. We conducted yet another unsuccessful round of calls and decided to expand recruitment to four neighboring municipalities. Again, we recruited no new interviewees.

After these elaborate efforts (in January-February 2022) we had recruited five interviewees. Three of them withdrew prior to the actual interview with reference to a heavy workload. In the second four-week term of the project, in May 2022, we conducted yet another round of calls to GP offices in the greater Oslo area and recruited three new interviewees. Considering the low success rate of our time consuming and persistent recruitment efforts, we concluded that this was part of the important lessons learned from the pilot – and settled on the five interviewees that agreed to take part. Always intended as an explorative pilot, the limited number of interviewees does not prevent the study from providing important insights and lessons relevant for the development of a nationwide study.

There are several reasons why the GPs did not prioritize participating in this study: First, is the extensive workload that they reported on, and which we know from research and media coverage – referred to above as the GP crisis. Refusals or non-responses are largely believed to be due to GPs necessary sorting of all inquiries they receive into urgent, less important, and unimportant tasks, where the patients come first and second, and inquiries such as those from us fall into the latter category. There is no room for surplus projects in a busy everyday work life. Many inquiries were probably filtered out by the secretaries before reaching the GPs. It is also conceivable that GPs perceive the topic as important, but their own experience as too limited - or they questioned what they would benefit from participating.

### Interviewees

The five interviewees were all experienced GPs who had worked more than 20 years in general practice. Two women, three men, four of which had their practice in districts in Oslo West. However, three of these practices were in the district of Frogner –which also include downtown Oslo, an area that has a high share of immigrant populations. While one GP reported she had a ‘very white, very middle class’ list (GP5), two reported that about 10% of their patients were immigrants or had non-Norwegian ethnic backgrounds (GP2 and GP4). One GP estimated that share to be around 40% (GP1) while the last GP had a large share of immigrants on his list and stated that he dealt with torture victims often (GP3). Four of out of the five interviewees confirmed that they had patients who they knew had survived torture on their list. While one GP stated that this was a common health issue in his practice, the others knew of less than five patients on their current lists. They did not, however, rule out that they had several torture victims among their patients without knowing. One GP knew several patients who lived with family members who had been tortured.^5^

All GPs were above 50 years old. None had a particular interest or qualifications pertaining to torture or refugee health.

### Interviews

Four interviewees preferred to conduct the interviews *via* zoom, which we accommodated – both due to the corona situation and to be flexible given their work pressure. One interview was carried out in the GP’s home office, with no other persons present. Anpalagan and Fesseha conducted all interviews together. Interviews lasted between 43 min and 61 min. All interviews were transcribed verbatim immediately after the interview, allowing for learning from one interview to the next. This meant that although we used the same interview guide for all interviews, our mode developed as we (AA and HF) grew more comfortable with the role as interviewers and with allowing silences to stir further reflections. The interview guide had two parts, the first consisting closed-ended question about the interviewees’ background (years of experience in general medicine, education, list size, share of patients with immigrant background). The second part consisted of open-ended questions asking the interviewee to reflect on the meaning of torture as they understood the term, give anonymized, illustrative or typical examples of patients with torture experiences, if and why they suspected torture injuries, how they would typically work to follow up on such concerns, their referral practices, and experiences with cooperation -and information exchange across health services. The interview guide was designed according to our interest in a broad understanding of GPs reflections about and practices vis-à-vis potentially tortured patients. While each interview covered the same general topics, the interviewees were not asked the same follow-up questions – these varied with interviewees and their weighting of different topics. Interviews were recorded, translated verbatim and then the recordings were immediately deleted.

### Analytic process

The results are based on a thematic analysis of the interview data. Put simply ‘thematic analysis is a method for identifying, analyzing and reporting [themes] within data’ [46]. Themes are understood as articulations of patterns and shared meanings within and across the data material, produced by the researchers at the intersection of the data itself, theory, and coding practices [47]. This means that themes are not developed in a vacuum. For this study, our attention was influenced by the study purpose, by the interview data, by the framing of the data provided by the interview guide, and the authors’ knowledge about health professionals’ organization and practice vis-à-vis potentially tortured victims in other parts of the health care system [10,48]. We read and reread the transcripts, and coded all interview data, initially indiscriminately with the use of on-the-spot keywords that reflected the various topics addressed or thematized in the given sentence or longer extract. Next, all keyword codes were grouped together in larger category themes and divided into primary and secondary codes – where primary codes eventually became themes for this analysis, and secondary codes reflect variations of ways in which the given theme is addressed. This process was recursive. We went back and forth with codes and keywords, placing them in different categories, renaming categories, and taking notes for each code, extract, and interview summary along the way – and agreed on broad, aggregated themes that captured the interview data. In this article we present our findings pertaining to themes that captured the interview data’s aggregated reflections on the prospects for identification of and care for patients who may have survived torture, focusing on interviewees’ reflections both as individual GPs and their meetings with patients, and as members of the broader public healthcare organization. We have removed filler words and adjusted some quotes softly for readability during translation from Norwegian to English, but without altering the meaning. We indicate the share of interviewees that held similar view or reported corresponding experiences for transparency, not generalizability.

## Results

We interviewed five experienced and dedicated general practitioners in Oslo, to explore their perspectives on, knowledge about, and practices pertaining to patients who had (potentially) survived torture. The findings are presented below in the main themes we constructed during the analysis: first we present the GPs reflections pertaining to conditions for identification of potential torture victims to take place. Second, we address the perceived advantages and challenges of general practice vis-à-vis this patient group that our interviewees raised. Lastly, we address their reflections on the organization and division of labor in the Norwegian health care system – including training and education – pertaining to identification and rehabilitation of torture victims. The main themes indicate different levels: the first primarily concerns individual knowledge and practice, the second captures profession-specific aspects, and the latter main theme addresses issues to do with structure as presented and indicated in interviews. Importantly, themes and subthemes are interconnected, not mutually exclusive.

### Individual practices for identification

The first step to rehabilitation, is identification. For several reasons, and as elaborated above, GPs may present torture victims with the first perceived possibility to share torture experiences with someone in a position to offer or secure adequate health care. But for that sharing to take place, the GPs underlined the importance of displaying a genuine human interest, and of treading carefully, in step with the patient’s pace. Identification cannot be forced or rushed, and the following quote from our interview with GP3 illustrates the stakes involved, also in general practice:

So, people come here, they come to a foreign country, they don’t know the language, they don’t know the culture, they don’t understand our norms or customs, and then someone also starts asking you if you’ve been raped or tortured, and… I believe a lot of people don’t want to confirm that kind of question. We are not wired that way, we don’t easily offer our vulnerabilities, the most degrading things we have experienced. (GP3)

GP3 went on also to emphasize that GPs need to be aware that many torture victims have poor experiences with health personnel who have ‘answered to dictatorial regimes rather than to the Hippocratic oath’, adding a barrier to trust between patient and doctor. GP2 formulated the meaning of trust concisely, summarizing what all our interviewees made a point of:

I think the same applies [for this category of patients], as in general, that in order to be competent in meetings with patients, you must begin with building the relationship, ask open questions, and be sensitive to whether you have established a foundation of trust, before you possibly proceed with specific questions (…) An important point is that you are on the lookout, perhaps listen extra carefully if there are any signals there, in what the patients say.

This listening skill is important, most of the GPs pointed out, because identification is rarely a straightforward process. According to our interviewees, identification is a gradual process where patients must be given time to feel safe and to trust their GP, before they are able to share painful experiences. This process is often lengthy, and two of the interviewees explained that torture victims are often also burdened with shame and guilt. As GP3 explained as well, this requires listening skills:

In many consultations, I can sense that there is something between the lines, and I carefully bring it up, and then I learn that they have not told this to anyone, ever. It can be a well-hidden secret that they cannot share, not with their parents, not with their spouse, not with their siblings, not with anyone. Because what they have been subjected to, is so f***ing horrible, but also because they also feel guilt. So, you must be sensitive, you have to be pretty good at communication to penetrate what this is really about. Then you also have to respect the fact that the patient can’t just open this book, they often can’t, but if you manage to build up a sense of trust, it really is amazing what comes up in the doctor’s office.

Importantly, one of the physicians cautioned that the symptoms that many patients with torture experiences present with can be rather easily dismissed or perceived as less serious than they are:

… a lot of the symptoms these patients present with involve a very high degree of somatization, that is, they have a lot of musculoskeletal problems and things like that, and if you do not know what they have been through, you might confuse their talk about these symptoms for … well… whining. Its rather easy to fall into that trap. (GP1)

The GPs also emphasized how many men had a hard time acknowledging mental health issues in general, and specifically, seeing that the torture they had survived had caused mental illness. The need for careful attention to underlying causes was explicitly articulated in GP3’s presentation of a typical torture victim vignette:

It is often complex, people come with backaches and headaches. But what is a headache? It’s a symptom. Or if you come and say you feel nervous and restless, what is that? It’s a symptom, it’s a headline: What’s behind it? Often, the explanation is traumatic experiences. There always is a reason. You can’t look at depression and anxiety and eating disorders and PTSD as if they live their own lives, you know? It’s connected to the life, the history, of that patient. What does he or she carry in their backpack?

GP4 further specified the need to convey to patients at the very start that ‘whatever your story might be, I can take it, and you are safe’. GP3 also highlighted the importance of explaining the professional duty of confidentiality thoroughly, citing ‘lack of respect for this obligation or lack of understanding of its implications in some other cultures’.

While all GPs emphasized the meaning of patient-doctor trust, the interviews brought up some assumptions about identification, too. One GP assumed that torture victims were identified in the asylum process: ‘I do expect that most people with that kind of experience are identified before [municipal resettlement]’ (GP 2). Another GP had established patients with torture experiences on her list who had shared their experiences without much facilitation on her part. On that ground, she appeared to expect that torture victims would share their experiences rather easily:

I have not [suspected that patients have been] subjected to torture, but I have suspected that patients have been or are subjected to abuse (…) I have experienced that several times, and then it has usually taken one or two years before they can even talk about it (…) If you have been subjected to torture, then I think it is a little easier for people to share that, because then it’s absolutely clear that they’re innocent. If they’re subjected to abuse, this is so shameful that they would rather not admit it, or the only way to survive is to pretend it doesn’t happen. (GP5)

Importantly, our interviewees highlighted gendered differences unsolicited, both in terms of torture experiences, and in patients’ health literacy and ways of dealing with and understanding associated health challenges. One interviewee pointed out that men are typically exposed to physical abuse, referencing a patient of hers that had been beaten with canes on the soles of his feet, crushing his bones and resulting in permanent challenges with walking,^6^ while women are typically subjected to rape and sexualized violence (GP4). GP3, somewhat to the contrary, emphasized how a lot of torture is sexualized in form, and that it does not exclusively target women. He added that male rape adds the stain of homosexuality, which in some contexts can be ‘the absolute crudest way to degrade people’. They both underlined that identifying victims of sexual torture require an extra level of trust and particular skills on the part of the health care professional.

Noticeably, none of the interviewees referred to the national guidelines for *Health services for asylum seekers, refugees, and family reunifiers* as they reflected around identification practices and conditions for sharing traumatic experiences, and none knew about the Istanbul protocol. But it is not necessarily reasonable to expect from generalist medical professionals to have an overview of all relevant guidelines and conventions pertaining to torture victims and injuries, given the proportion of patients that they know of who have survived torture.

In sum, the interviewees illustrated the importance of timing and sensitivity, trust and listening skills in order to identify torture victims in consultations at the GP office. Some of the interviews also put forward assumptions about identification in the asylum process and about how easy it is to share torture experiences relative to other forms of abuse that are not supported by either research or policies. They all appeared confident in their approach and role, but also pointed out that their many years in general practice was key to that.

### Profession-based pragmatism and the continuity of care

Several of the GPs interviewed for this pilot emphasized that torture victims are relatively more demanding as a patient group because of the extra time, patience and trust needed. Patient time builds this trust, a precondition for identification of torture injuries – as established by both research and accounted for by our interviewees [49]. In this regard, they made several nods during interviews to the GP-crisis, and pointed out how too long lists and far too short consultations do not allow for in-depth digging into the causes of symptoms that potentially tortured patients present with. As one interviewee explained, many (younger) GPs have too many consultations a day, ‘and don’t have time to ask. It’s fast in, fast out. And the question is: how much do you get to share in a 10-minute consultation?’ (GP5).

However, an important common characteristic of the interviewees that participated in this study, is their decades-long experience as GPs. While all of them referred to and acknowledged the above-mentioned GP crisis, they pointed out how the time pressure referred to as a severe consequence of the crisis did not apply to them. Because they were experienced, they knew most of their patients well after many years as their GP, which made their patient lists and workload manageable. They prepared consultations and set aside the time needed for patients with complex histories or health challenges – a coping strategy that was contingent of them knowing their patients well. As several remarked, the GP-scheme with 20-minutes consultations per patient, is not really designed for torture victims in terms of the time available. GP2, for instance, emphasized how setting aside plenty of time for consultations with torture victims was a conscious way of signaling ‘I got time for you, we will take the time that we need to talk about this’. Another interviewee explained how she managed her list for patients who had just started to share traumatic experiences:

You can’t open a box full of emotions and then just close it again and tell the patient to wait four weeks until they get another appointment. That’s of no use. So, you simply have to somehow make room for them to come back and continue the session, preferably a double consultation after two days. You cannot wait very long, and when they see that you prioritize them, it does something to the relationship you have with the patient. (GP4)

Provided manageable lists, general practitioners get to know patients in ways that are rare in other parts of medicine, to the benefit of their diagnostic work and patients’ health in a life course perspective. GP5 commented that it is her job to make sure ‘they know where to go whether it’s their throat or their soul that hurts’. In some ways, the quote illustrates general practice as a kind of holistic practice – the GP has a low threshold for contact, and sees, cares for, and gets to know the whole person that the patient is, not only the specific symptoms addressed during one consultation. They can make assessments based on a complex picture that involve e.g. work conditions, the status of close relationships, and health situation over time, in addition to clinical findings. As GP4 stated, ‘in many ways, it is this conversation that is our primary tool’. The continuity of care also relieves patients of having to tell their stories time and again, as GP3 pointed out:

I’ve known some of ‘my’ torture victims for many years – they know exactly where they got me, and vice versa. They don’t have to start at the creation of the universe every time we meet – because that, too, can be like hell. If you are referred to specialist health care, you must start again at the creation of the world, and again every step of the way. (…) Continuity really is one of the best things about general practice and medicine, and it is not well taken care of in the specialist healthcare services.

While the interviewees had found ways to make their patient lists manageable, three still considered tortured victims a demanding group of patients in terms of the time needed to invest in consultations and follow-up. With predictability, this group of patients would need longer consultations, and as GP5 explained:

You have to be very patient. Of course, for me, it is easier if someone comes just for birth control pills or earache – compared to patients who present with a very complex dysfunction. That does take a lot of detective work.

However, weighed against actual needs and experiences the time aspect and patience required is minor, GP4 commented:

I don’t really think they’re that demanding. In general practice there are a lot of people who complain about small things, all the time. Those who have been tortured, they have real traumas that they have to work through, and in view of their sufferings, I think that many of them are not in the whiny category, to put it that way.

In sum, the participating GPs appeared confident in their abilities to meet torture victims in a sensitive and good way – and in the value that is patient-doctor continuity in general medicine. In terms of patient list size, the five GPs’ average list size was above the national average, indicating that time management in general practice is also a matter of experience and getting to know the patients, not exclusively the number of patients on the list. In some ways, their accounts of how they meet torture victims in their practice appear to live up to the motivations for getting into general medicine in the first place: the continuity of care and trust between doctor and patients [17]. However, the GPs also pointed out challenges in their practice pertaining to this patient group in particular – most of which had to do with the broader organization of the healthcare system, which we turn to next.

### Limited resources and organizational gaps

All interviews addressed the structure and organization of the Norwegian healthcare system. Usually, the GPs brought it up as they reflected on their own experience or practice vis-à-vis potentially tortured patients. If the interviewee did not address the topic on their own initiative, we included a final question that asked them to reflect on the organization of the Norwegian healthcare system in terms of its ability to address the needs of patients who have been tortured. Overall, the interviewees’ concerns pertaining to the organization and structure of the health care system were three-fold, and pertained to a lack of coordination between sectors and across services, gaps in their education pertaining both to torture and cultural competency, and limited capacities in specialist health care.

Four of the informants pointed out that the healthcare system is inadequately organized to deal with victims of torture. The fifth interviewee held that the organization and structure as such is fit for purpose, but that the problem vis-à-vis torture victims and others in need of specialist psychiatric healthcare, is a particularly poor capacity across the psychiatry field (GP2). He also expected that torture victims were identified prior to their municipal re/settlement, as accounted for above, which is an implicit critique of the lack of systematic identification measures and a lack of coordination and cooperation between sectors. GP1, too, commented that if he was the one who identified and referred torture victims to specialist healthcare, that meant that the victim had already been in Norway in what probably amounted to years. He continued: ‘This should have been secured upon entry, at least during the first six months’.

The interviewees had practiced medicine for decades and could not remember whether torture sequelae were covered in their university training. But as GP4 commented: ‘We did not learn enough to be prepared to meet this group of patients’. GP3 argued that the professional studies in medicine now ‘needs to start from the fact that Norway is a multicultural society, there is no reason to discuss it anymore, [the question should rather be] how we should handle it and approach it as professionals’. He underscored the meaning of cultural competency and sensitivity in his work, and the need for better training in med school and specialization courses, but also added that there is a limit to how deep into these matters a generalist should be expected to go. As he summarized: ‘All physicians cannot be social anthropologists’. GP1 added further shortcomings in his continued medical education options:

Here, there is a lot of diabetes, a lot of cardiovascular diseases, a lot of metabolism, a lot of gynecology, a lot of orthopedics, surgery… There has also been a lot of substance abuse courses in recent years within psychiatry. But maybe we should have more courses on refugee health.

Their reflections and experiences resonates with recent research, as accounted for in the introduction: there is limited focus on torture in Norwegian medical training and education, and little information sharing across sectors [10,11]. The interviewees turned most serious, however, when they addressed the capacity and quality of services in specialist health care. In Norway, the main responsibility for specialist psychiatric healthcare services is organized in District Psychiatric Centers (DPSs). GP1 commended the local DPS for their quick handling of referrals, for taking patients seriously, and for transferring thorough journals and medical case epicrises. The other interviewees, however, referred to an overworked and underpaid field of psychiatry. GP4 criticized the information flow from DPSs to GPs as too brief and too slow, hindering her work as a GP for their common patients. GP3 was cautious about referring patients to the DPS unless he knew who would treat the patient: ‘With all due respect, if you refer your patient, she or he is at risk of coming to someone who is still under education, or who has just hatched (sic!), someone who is not yet a specialist, or who is practically a psychology student…’ GP2 on his side was convinced that DPSs, due to unbearable workloads, deliberately returned referrals for more detailed references ‘which is not clinically justified, but rather a delaying tactic, I feel’. Commenting on referral practices and quality of services, GP3 went as far as to say that when he thought of the Norwegian healthcare system, he didn’t even include psychologists and psychiatrists outside of public hospitals – because they were completely unavailable, with year-long waiting lists.

In extension of their critique of the (availability of) specialist healthcare services, three out of the five interviewees brought up a need for specialist centers for victims of torture, with employees who are proficient and specialized on refugee health, war trauma, and torture specifically. As one GP reflected: ‘It may well be that there should be a separate specialist center for those who have been exposed to such things. Perhaps that would have made more sense’. (GP4) GP5 commented on what she saw as an untimely development:

When I first started as a general practitioner, there was a center for refugees and victims of torture, that has since been closed… So even if the problem has actually increased, the services are reduced, a bit in the wrong way.

She explained how as a GP you can meet a new torture victim once every five years – which does not really equip you with the weight of experience, nor legitimize the prioritization of in-depth study of this particular patient group among many other conditions. That, however, was not to say that the patient group is not sizeable enough to warrant expertise. As she said: ‘it would be good to gather such expertise at a specialist center, with professionals who would then meet this patient group often and serve as a prepared and qualified network to lean on and refer to’.

In sum, the GPs addressed shortcomings in identification measures prior to re/settlement, in education and specialization training, and, most fervently, expressed their concerns about what they considered to be severe capacity limitations in necessary specialist healthcare services. They also lamented the lack of a specialist referral center with whom to consult in need.

## Discussion

In the following we will address three critical and interrelated aspects that were raised in and by the interviews that corresponds to the three main themes in the result section: 1) commonsensical reasoning on identification; 2) professional confidence in general practice; and 3) structural challenges to qualified health care for torture victims in the Norwegian health care system.

### Commonsensical reasoning on identification

As stated, the Norwegian Directorate of Health expect all health professionals to know the symptoms of torture and how to follow up on identification and follow-up according to international guidelines. Our pilot indicates that health policy as written and health policy as performed by healthcare providers at the frontline differs when it comes to identification of potentially tortured patients, and that knowledge and practices also vary between professionals. While several studies and practitioners (including ours) highlight the need for more knowledge and competence on torture and refugee health more broadly [10,50], the risk of commonsensical and wrongful reasoning in its absence is rarely addressed. While the five experienced GPs that participated in this study demonstrated great care for their patients, the interviews also drew attention to commonsensical assumptions pertaining to identification of torture victims. The first: that torture victims are identified prior to re/settlement and entry onto GPs’ patients lists. While this assumption was only explicitly articulated by one of the interviewees, we have met this expectation numerous times while working on this topic. Peer students, colleagues, legal scholars, and physicians in our networks find our focus on identification practices within general practice peculiar. They assume questions regarding torture and inhumane treatment to be explicitly on the agenda in asylum interviews and that victims will account for their experiences early on as it will support their claim for legal stay. Such commonsensical reasoning appear intuitive as the risk of torture upon return does provide a right to protection and asylum [24]. It also indicates a potential coping strategy, in that it relieves the professional of the need to ask about and take the time to process and address traumatic experiences, as it shifts the responsibility of doing so to other sectors and public servants. It falters, however, on closer inspection, as illustrated by the research that stirred this pilot: Many torture victims go through the asylum process without being identified – not for lack of rehabilitation needs, but for lack of trust in authorities, based precisely on their experience with torture, and because – contrary to common assumption – they are not systematically asked about experiences with torture or other inhumane treatment upon arrival [9–13].

The second commonsensical reasoning about identification is related to the first. While most of the interviewees emphasized that patients found it difficult to share their torture experiences, even after many years, one GP explained that she expected it to be ‘a little easier’ for torture victims to share their experiences than, say, domestic abuse victims. Her take was probably based on her experience with established victims of torture who had shared their experiences with her in a more straightforward way. However, this kind of reasoning is not supported by substantial research that addresses barriers for torture victims to share their experiences. Typically, such research highlights the lack of trust in authorities and strong feelings of guilt and shame [51,52] as factors ‘that discourage disclosure in health care services’ [18]. Several of the interviewees also commented on how sexual torture probably were more stigmatized than non-sexualized forms of torture, raising the barrier for disclosure.

Combined, there is a concerning risk that patients’ torture experiences are not identified because of an expectation that they are already identified and catered to or that they will share their experiences unsolicited. Matched with a strong professional confidence, such commonsensical reasoning may add an extra barrier to proper and timely identification of torture victims and injuries.

### Professional confidence

As stated, the Norwegian Health Care Services Act [37] emphasizes that health services are to provide universal health care, equal access and quality of services in primary care. As street-level bureaucrats and frontline workers, GPs need (and want) to deliver sufficiently good services to each individual patient. It is a paradox, thus, that time – key to patient care and trust – is both the specialization’s most valuable asset, and also particularly short in supply in Norwegian general practice [17,32]. The principle of equal services can become difficult to comply with when time is in short supply [40]. Importantly, however, our GPs reported that they now had manageable lists with patients they knew well, an accomplishment they ascribed to their seniority and experience. They emphasized how this enabled them to set aside extra time for patients they knew needed it – a pragmatic, or street-smart, approach to the management of their patient lists. Moreover, the continuity that comes from knowing patients well and over time enables more holistic care and is likely to save time in the long run – as the GPs do not have to build trust and get to know patients in each consultation in order to recognize and identify the origins of symptoms or histories that are medically relevant but difficult to share.

Interestingly, previous research has documented how GPs can feel uncertain about their clinical decisions vis-à-vis refugee patients more broadly [33,34]. In somewhat contrast, the interviewees in our study appeared confident about their level of knowledge and comfortable with their uncertainties. Some also demonstrated a particular sensitivity to patients with torture histories – as they both set aside extra time to communicate to patients that they were prioritized, and emphasized the need to reassure patients that whatever their story was, they could take it. Additionally, while recent research finds that the gendered dimensions of torture is critically neglected both in research and practice [53,54], the participants in our study demonstrated a particular attention to the matter, and brought this to the interview agenda themselves. They pointed to the stigma associated with sexual violence and torture for both men and women, and to gendered aspects of health literacy particularly pertaining to mental health issues. Moreover, they compared torture and sexual torture to other forms of sexual violence in peacetime. One interviewee also challenged the distinction between torture defined as state-perpetrated violence, and the sexual violence that many refugees are subjected to as they seek a better, safer life for themselves and their kin – and – implicitly – the hierarchy of suffering and rights indicated by that distinction.

Overall, the interviews illustrated a seasoned professional confidence, that also illustrated their sensitivity towards and ability to meeting patients where they are at. Perhaps most importantly, the interviews demonstrated how – when the pressures of the GP-crisis is out of the question as for our interviewees – general practice provides the platform to develop patient-doctor relationships that emphasize the whole person that the patient is, genuine, professional interest, trust, time and continuity, which are factors conducive to patients’ sharing of vulnerabilities and torture or torture-like experiences. The interviews suggest that for conditions for identification to be right, GPs need the time, flexibility, patience, and continuity that manageable lists allow for.

### Structural challenges to qualified healthcare for torture victims

According to the national guidelines for *Health services for asylum seekers, refugees and family reunifier* [28] the disclosure of health challenges and injuries related to torture after the arrival phase and asylum process ‘shall generate relevant investigation and documentation’ according to which competent health personnel must assess the need for further follow-up of any disclosed injuries, and ‘how this follow-up should be done’. Yet, recent research establishes that torture is overwhelmingly absent in the curriculum of professional studies in medicine in Norway, as also our interviewees expressed concern about [10]. Moreover, our interviewees were unfamiliar with the Istanbul protocol and the guidelines for follow-up should patients disclose that they had been tortured. Importantly, they had several concerns about the availability and quality of and cooperation with the specialist health care services that they are gatekeepers for [16]. While they acknowledged the GP-crisis, they were far more concerned about what could be labelled a corresponding ‘DPS-crisis’ – i.e. the de facto unavailability of public, specialist psychiatric healthcare services when needed [55,56]. These structural limitations necessitated the GPs’ exercise of professional discretion. For some, this resulted in hesitation to refer to specialist health services altogether – as they did not trust the quality of services their patients would receive and because they expected year-long waiting lists. In response, they rather did their best to cater to their patients’ needs themselves.

Given the decades of practice they represented – all our interviewees begun their careers as general practitioners more than 20 years ago – they started to practice general medicine in a system that included what was called *Psychosocial center for refugees.* This center was a psychiatric outpatient clinic in Oslo and neighboring districts, available upon referral for adult refugees with psychological problems following torture and/or other traumatic experiences. The center was also tasked with competence-building, teaching, and peer guidance, as well as research based clinical activities. The center closed its doors in 2004 against expert advice [57]. Several of our interviewees lamented the absence of such capacities and pointed out the irony in the downscaling of specialist healthcare services for refugees amidst an ongoing refugee crisis and an increasing population with refugee background. Important in this regard, as one of our interviewees stated, the number of torture victims on each individual GP’s patient list is too small to provide every GP with the weight of in-depth knowledge and training from experience. Considering the already limited capacity of the DPSs and the lack of torture specific specialist knowledge across medical professions, the re-establishment of a referral center that specializes on torture rehabilitation and refugee health more broadly could function as a go-to resource to call for advice when refugee patients present with complex trauma histories. Recent and ongoing conflicts and wars in and beyond Europe results in an increasing refugee population with potential torture trauma histories, and are likely corresponds to an increased need for specialized healthcare services both for rehabilitation referrals and immediate advice.

## Conclusion: limitations and implications

Healthcare policies can strutter with ideals of equality, equity, and universality. But it is the everyday work pragmatics of frontline workers navigating between rules and regulations, limited resources, long patient lists, and individual patient’s needs that make health policy in practice, and hereby directly affect the lives and healthcare of patients. While indebted to research on the health impacts of torture and the quality and requirements of specialized healthcare for torture victims, access to specialized services is, in many contexts, contingent on proper referral by general practitioners. We do not know to what extent torture victims are identified by GPs, how they are met, or what GPs know about torture, identification, and proper response. This article has presented findings of a recent pilot study that addressed this knowledge gap in the context of the Norwegian healthcare system.

In the analysis, we have used SLB vocabulary to approach GPs acoounts of their practices, discretional prioritizations, and potential coping strategies in encounters with complex and extraordinary trauma. Our research indicates that GPs can be important actors in terms of identifying torture victims after re/settlement, but that there are also shortcomings in their training, in the overloaded GP-scheme, in approaches to identification, and in specialized healthcare, that limit prospects for identification and thereby also rehabilitation at present.

Our pilot study has several limitations as well as implications for future research:

### Limitations

We did not define torture for our interviewees, but rather asked them what they understand the term torture to mean. Hence, some of the interviewees defined torture broadly. For instance, one interviewee addressed how migrants may carry experiences that resemble torture, although the violence is not perpetrated by state forces as required per the Torture convention, such as refugees that cross the Mediterranean, and the ‘sexual abuse [that] is hidden in that transport?’ (GP3). Some likened the experiences of refugees and migrants who had been tortured to the experiences of patients who lived through domestic violence. While we were careful to emphasize that our focus was on refugees with torture experiences, we cannot be certain that all five interviewees distinguished clearly between torture, torture-like experiences, and other forms of trauma and/or violence in their reflections. The limited number of interviewees and their rather similar profiles in terms of experience constitute an important limitation of this study. Overall, we believe that the consistent findings across interviews reflect this bias in recruitment, rather than GP experiences more broadly. The observation that all interviewees have more than 20 years of practice as GPs in combination with our extensive recruitment efforts suggest that younger generation GPs do not prioritize and/or do not have the time to participate in research. When their everyday work life does not allow participation in research, we lose out on valuable perspectives that could contribute to improved conditions for both the patient group and GPs. It is difficult to build knowledge about a professional group’s practice, work conditions and knowledge without getting professional practitioners engaged in research. As a pilot, however, we never intended for the study to be generalizable. The limitations are, we hold, outweighed by lessons learned and the study’s contributions to the tuning in of issues to be aware of and questions to explore further in later research.

### Implications

With the ongoing, brutal wars in mind, the need to identify and secure rehabilitation for torture victims is likely to increase in the near and foreseeable future, in Norway and beyond. Knowing that many torture victims are not identified upon entry as asylum seekers, refugees or for family reunification, GPs can play a key role in this task and help states live up to their obligations as members to the UN Convention against torture. However, more research is needed. Future research efforts need to be flexible and innovative in order to recruit broadly from the GP profession. Granted that future research manages to recruit from across the profession, it should explore how perspectives, knowledge, and practices – including referral practices – vary with generations, geography, socioeconomic background, and training. SLB theory can be used to understand discretionary practices, differences between GPs’ choices, and if and in what ways attitudes of GPs influence them in their work. It is noticeable that none of the interviewees mentioned the municipal refugee health services that are increasingly established in municipalities in Norway. While their expertise is not pertaining to torture and trauma specifically, their role and use as potential expertise by primary health care professionals should also be examined.^7^

Should future research confirm the findings in this pilot, it should carry implications for the curriculum in education and continued medical education training pertaining to torture, for cross-sectorial cooperation and communication, and start a conversation about the need for qualified and available expertise in torture rehabilitation.

## Supplementary Material

Thematic interview guide_AFH.docx

## Data Availability

The data used and analyzed for this article are deidentified and stored on a secure drive at the University of Oslo. The transcripts are not made publicly available due to the sensitive and personal nature of the information contained and to secure the anonymity of the interviewees. The anonymized data are available from the corresponding author upon reasonable request.

## Notes

1 While that may not seem like an alarming number, for comparison it is, for instance, almost triple the number of patients diagnosed with e.g., leukemia, and 50% more than the number of patients diagnosed with Type 1 diabetes (see e.g. Kreftregisteret.no and Helsedirektoratets National Guidelines for Eating Disorders).

2 In Sweden, the Red Cross operates specialized rehabilitation services for traumatized and tortured refugees in collaboration with authorities https://www.rkh.se/en/research/swedish-competence-center-for-rehabilitation-of-torture-and-war-trauma/, accessed March 17, 2023. Denmark has a specialized rehabilitation service for refugees that includes torture injuries–provided by private hospitals incorporated into the public health services. Access is free, contingent on GP referral and residence permit. See, e.g., https://www.dignity.dk/en/rehabilitation/, accessed March 17, 2023.

3 As per 2022, according to the municipality of Oslo: https://www.oslo.kommune.no/statistikk/befolkning/befolkningsutviklingen-i-oslo-gjennom-2022, last accessed March 18th, 2023.

4 As per overview of Oslo at the National online health services in Norway, https://tjenester.helsenorge.no/bytte-fastlege?fylke=03&kommuner=0301, last accessed March 18th 2023.

5 It is well-established in research that the traumatic experiences of torture victims and refugees can also have severe mental health consequences for their families and the next generation (41, 42, 43).

6 This is a widespread form of torture referred to as falanga/falaka/bastinado (47).

7 Thank you to the anonymous reviewer who pointed this out.

## Author details

Department of Interdisciplinary Health Sciences, Institute of Health and Society, Faculty of Medicine, University of Oslo, Norway.
