# Supplementary material for: General practitioners’ knowledge and practice in consultations with (potential) torture victims: a qualitative pilot study from Norway
Source: Scand J Prim Health Care. 2024 Sep 13;42(4):723–37. doi: 10.1080/02813432.2024.2404054 (PMC11552259; doi:10.1080/02813432.2024.2404054)
Supplement: Thematic interview guide_AFH.docx [file IPRI_A_2404054_SM7245.docx]

**Thematic interview guide**

(translated version – all interviews were conducted in Norwegian).

**1. Practical information**

1. District
2. Size of patient list
3. Number of years in general practice
4. University
5. Diversity of the patient group (estimated share of patients with immigrant background)

**2. General knowledge**

1. How do you understand the term “torture”?
2. What are your immediate thoughts, as a GP, when we say torture and torture victims/patients?
3. Have you ever suspected that a patient of yours have been tortured?
4. How would you proceed if you suspected that a patient had been tortured, or have you ever been in such a situation?
5. Do you know what rights tortured, resident refugees have in the health care system - and relevant guidelines for care?
6. Do you know any guidelines for identification/ investigation/ treatment (ie the Istanbul Protocol)? If yes, how did you learn about these?
7. If you have/had felt insecure about how to address torture injuries or victims, who would you consult with?
8. As a GP, where do you think or feel that your responsibility as a GP starts and ends for this particular patient group (vis-à-vis other parts of the health services or other sectors)?

**3. Experiences from practice**

1. How would you present a 'typical' patient who raised your concern that s/he had been tortured? (gender, age, background, reason they scheduled appointment in the first place, etc.)
2. With specific patients in mind:
   1. Are these patients who have been in Norway for a long time?
   2. Have they had health problems as a result of torture for a long time?
   3. Have they received help for the symptoms without the cause having been identified?
   4. Do they tell about their experiences of torture on their own initiative?
   5. Does it take time for them to do so?
3. How would you describe this patient group? (in comparison with patients in general)
4. Do you do anything in particular to build a sense of safety and trust?
5. How do you experience the cooperation and interaction with specialist health care services?

***Culture and language***

1. Is knowledge of immigrant and refugee health and cultural competence important in your work? If so, in what way(s)?
2. How good health literacy and skills do you perceive the relevant patients to have?
3. Have you experienced that language can create a barrier that makes the anamnese/investigation/conversation difficult?
4. Do you use interpreters in consultations, and what are your experiences in the context of traumatic experiences? Issues to address:
   1. trust between patient and interpreter
   2. interpreters’ relevant language/linguistic skills and competence for the task
5. How do you handle and/or cater to relatives in consultations with potentially tortured patients?

**4. Organization of health care services/system**

1. Do you think that the Norwegian healthcare system is (sufficiently) organized to address the needs of patients who have been tortured?
2. Would you find a focused continued education course (for GP specialists) on how to identify and treat torture injuries and patients who have been tortured useful for your own practice? (including topics such as diagnosis, rehabilitation, specialist health services, rules and regulations, interpretation, refugee health, cultural competency)?
